# Butyrate and propionate inhibit antigen-specific CD8^+^ T cell activation by suppressing IL-12 production by antigen-presenting cells

**DOI:** 10.1038/s41598-017-15099-w

**Published:** 2017-11-06

**Authors:** Claudia Nastasi, Simon Fredholm, Andreas Willerslev-Olsen, Morten Hansen, Charlotte Menné Bonefeld, Carsten Geisler, Mads Hald Andersen, Niels Ødum, Anders Woetmann

**Affiliations:** 1 Department of Immunology and Microbiology, University of Copenhagen, Copenhagen, Denmark; 2 Center for Cancer Immune Therapy (CCIT), Department of Hematology, Copenhagen University Hospital, Herlev, Denmark

## Abstract

Short chain fatty acids (SCFAs), such as acetate, butyrate and propionate, are products of microbial macronutrients fermentation that distribute systemically and are believed to modulate host immune responses. Recent data have indicated that certain SCFAs, such as butyrate and propionate, directly modulate human dendritic cell (DC) function. Given the role of DCs in initiating and shaping the adaptive immune response, we now explore how SCFAs affect the activation of antigen-specific CD8^+^ T cells stimulated with autologous, MART1 peptide-pulsed DC. We show that butyrate reduces the frequency of peptide-specific CD8^+^ T cells and, together with propionate, inhibit the activity of those cells. On the contrary, acetate does not affect them. Importantly, butyrate and propionate inhibit the production of IL-12 and IL-23 in the DCs and exogenous IL-12 fully restores the activation of the MART-1-specific CD8^+^ T cells, whereas IL-23 has no effect. In conclusion, these results point to a pivotal role of butyrate and propionate in modulating CD8^+^ T cell activation via the inhibition of IL-12 secretion from DCs. These findings reveal a novel mechanism whereby bacterial fermentation products may modulate CD8^+^ T cell function with possible implications in anti-cancer immunotherapy.

## Introduction

The gut microbiota has strong influence on the human immune system and health via the production of a variety of metabolites detectable in host circulation^1–3^. Through fermentation of dietary poly- and oligosaccharides resistant to digestion in the small intestine, the distal gut microbiota can metabolize complex carbohydrates to produce small organic acids, the majority of which are comprised of the short chain fatty acids (SCFAs) acetate, propionate, and butyrate^4^. Acetate, propionate and butyrate are found at molar ratios of 60:20:20 in the intestinal tract, reaching peak concentrations in the gut lumen (50–100 mM). Due to their structure they are easily absorbed into the portal circulation, through which they reach the bloodstream at much lower concentrations than found in the gut lumen^5–9^. SCFAs affect cells both by the interaction with their specific G protein-coupled receptors (GPR)-41, GPR43 and GPR109A and independently of these receptors^10–13^. Butyrate and, to a lesser degree, propionate inhibit histone-deacetylases (HDAC) thereby affecting host gene expression^14^ and inducing autophagy^15^. Several evidences highlight the importance and broad role of SCFAs from regulating energy homeostasis^16^ to modification of immune cell function and responses^8,9,17,18^. We have shown that human monocyte-derived dendritic cells (DC) express GPR41 and GPR109A, and that SCFA treatment affect gene expression in lipopolysaccharide (LPS)-activated DCs^19^. In peripheral tissues, DCs are found in an immature stage specialized in detecting and capturing antigens through expression of innate pattern recognition receptors. When activated by pathogen-associated molecular patterns, immature DCs undergo phenotypic and qualitative changes to become mature DCs and migrate from the periphery into the draining lymph nodes where they present pathogen-derived peptides to CD4^+^ and CD8^+^ T cells^20–22^. The environment in which the DCs are activated greatly shapes their ability to activate and differentiate T cells^20^. Cytotoxic CD8^+^ T cells (CTLs) are pivotal for the killing and clearance of virus-infected cells and cancer cells. The generation of antigen-specific memory and effector CTLs from naïve CD8^+^ T cells relies on antigen presentation by DCs^23–25^ in combination with surface expression of co-stimulatory molecules like CD80, CD83, CD86, CD40, OX40-L or ICOS-L^21,26^. In addition, inflammatory cytokines like IL-1, IL-6, and IL-12 produced by the activated DCs and/or macrophages during the priming phase provide the necessary signal 3 that further induces cell division and development of CTL effector functions^27–30^. To study the effect of SCFAs on the DC-mediated activation of antigen-specific CTLs, we studied MART-1-specific CD8^+^ T cells co-cultured with MART-1 peptide pulsed autologous HLA-A201^+^ DCs previously treated with SCFAs. We used MART-1 as model antigen since around 0.1% of naive CD8^+^ T cells are MART-1–specific and they can be found in HLA-A201^+^ individuals regardless of prior antigen exposure^31,32^. We found that butyrate and propionate significantly suppressed the activation of MART-1-specific CTLs. In search for mechanism involved in this suppression, we discovered that butyrate and propionate inhibited production of IL-12 and IL-23 in the DCs and that the CTL activation could be fully reconstituted by exogenous supplementation of IL-12. Our work adds a new dowel to the understanding of the host-microbiota mutualism by revealing that SCFAs can dampen CTL activation via their effect on DCs.

## Results

### Butyrate and propionate inhibit activation of antigen-specific CTLs

To study whether SCFAs affect activation of CTLs in T cell-DC co-cultures, we used monocytes isolated from healthy HLA-A0201^+^ donors to generate immature DCs (iDC). The iDCs were either left untreated or were stimulated with LPS for 24 h in the absence or presence of sodium acetate, sodium butyrate, or sodium propionate to generate mDC, mDC_A, mDC_B, and mDC_P, respectively, as previously described^19^. After 24 h, the DCs were pulsed with peptides and cultured for 10 days with autologous CD8^+^ T cells.

**Figure 1:**
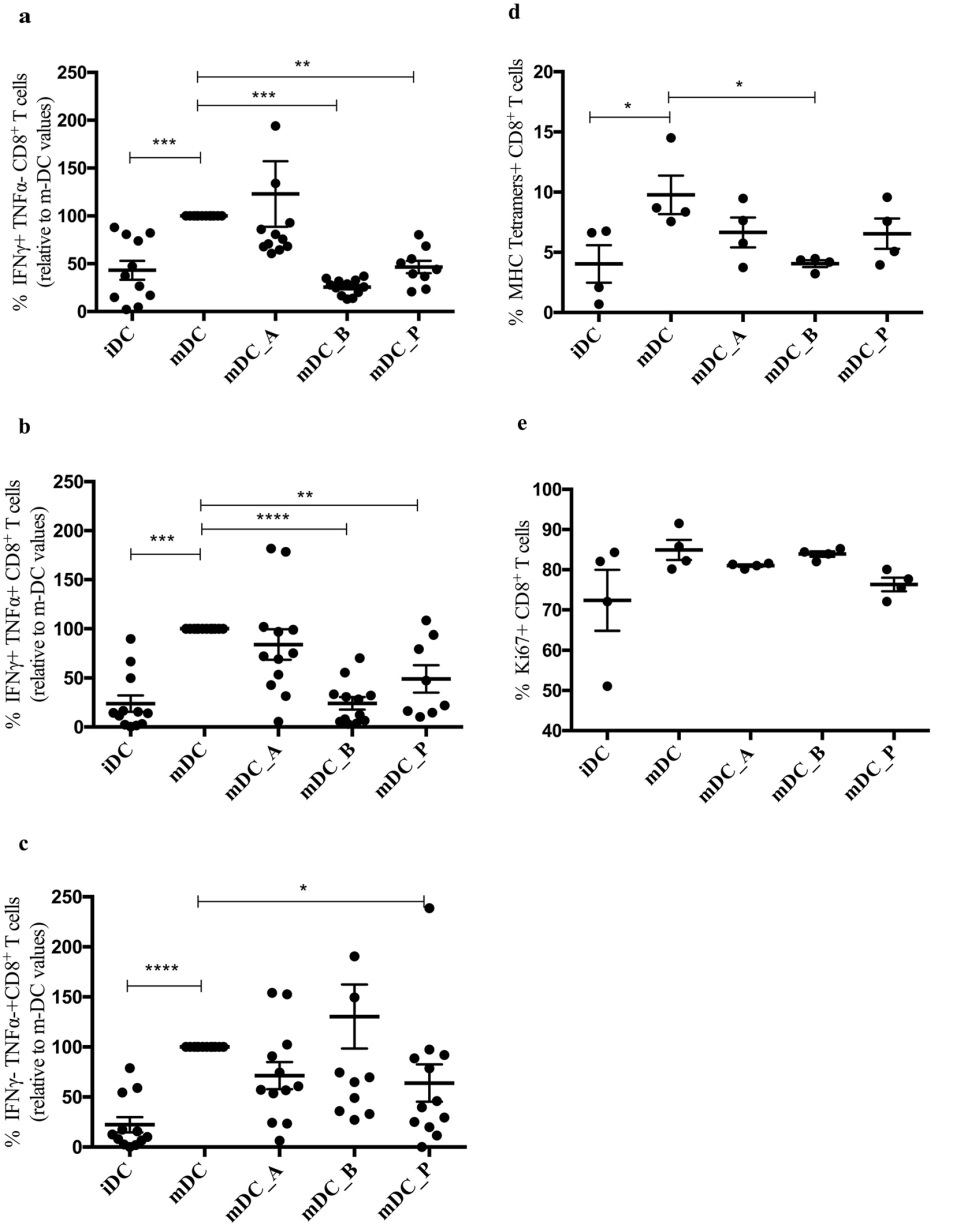
Butyrate and propionate inhibit activation of antigen-specific CTLs. (**a**) Chart represents the percentages of activated IFN-γ^+^ TNF-α^−^ and (**b**) IFN-γ^+^ TNF-α^+^- and (**c**) IFN-γ^−^ TNF-α^+^-producing CD8^+^ T cells after 10 days of co-culture with MART-1 pulsed iDCs, mDCs, mDC_A, mDC_B, and mDC_P. Values have been normalized to those CD8^+^ T cells in co-culture with untreated mDCs that has defined the activation threshold for each individual donor (n = 12). Shown are the percentages of (**d**) MART-1-specific CD8+ T cells and (**e**) KI67^+^ CD8^+^ T cells in co-cultured with DCs treated as previously mentioned (n = 4). Wilcoxon matched-pairs signed rank tests were run for the a–c analysis, and Mann-Whitney tests for d–e. **P ≤ 0.01, ***P ≤ 0.001.

Subsequently, we analysed the frequency of activated MART-1 specific CTLs as determined by IFN-γ^+^TNF-α^−^, IFN-γ^+^TNF-α^+^-, or IFN-γ^−^TNF-α^+^-producing CD8^+^ T cells. In order to reduce the variability among donors, we normalized the measured frequences of each subpopulation to the respective values obtained when CD8^+^ T cells were in co-cultures with mDC. We found that expansion of both the IFN-γ^+^TNF-α^−^ and the IFN-γ^+^TNF-α^+^-producing MART-1 specific CD8^+^ T cells was significantly impaired in mDC_B (P ≤ 0.001 and P ≤ 0.0001, respectively) and mDC_P co-cultures (P ≤ 0.01 and P ≤ 0.01, respectively) (Fig. 1a and b), while the IFN-γ^−^TNF-α^+^-producing CD8^+^ T cells expansion was only significantly impaired in mDC_P co-cultures (P ≤ 0.05) (Fig. 1c). In addition, non-normalized values were reported in Supplementary Fig. 1. Indeed, analyses of the raw data confirmed the strong inhibition of the expansion of IFN-γ^+^TNF-α^−^ and IFN-γ^+^TNF-α^+^-producing CD8^+^ T cells exerted by mDC_B (P ≤ 0.001 and P ≤ 0.001, respectively), followed by mDC_P (P ≤ 0.01 and P ≤ 0.05, respectively), that also has a significant effect on the IFN-γ^−^TNF-α^+^-producing CD8^+^ T cells (P ≤ 0.05) (Supplementary Fig. 1a,c). Essentialy similar tendencies were observed when we used a peptide from another weak, endogenous antigen (programmed-death ligand 1, PDL1; Supplementary Fig. 2a,b) or peptides from two different strong, exogeneous antigens (Epstein Barr- and Cytomegalo-virus, EBV and CMV respectively; Supplementary Fig. 2c–f), indicating that the inhibitory effect, primarily exherted by butyrate, on DCs ability to prime and activate CD8^+^ cells was not limited to MART-1 specific CD8^+^ T cells. Nevertheless, we found a strong consistency of CD8^+^ responses when MART-1 peptide was used, compared to the others. Given the low donor variation observed with MART-1 peptide, we decided to use MART-1 as model for further analysis.

To verify that the activated CTLs were actually peptide-specific CTLs, we stained the cells with HLA-A201/MART-1-tetramers. Expansion of MART-1 specific CTLs was significantly impaired in co-cultures with mDC_B (P ≤ 0.05), but not if in co-culture with mDC_P, when compared to mDC as control (Fig. 1d).

To check whether any of the SCFAs could reduce DCs induced CTLs proliferation, we simultaneously stained the CD8^+^ T cells for the proliferation marker Ki67, a nuclear protein that plays a pivotal role in the regulation of T cell division^33^. We found that the levels of Ki67 were similar for all the groups of CD8^+^ T cells regardless of the SCFA used to pretreat the DCs during their maturation (Fig. 1e).

Taken together, these results indicated that the SCFAs, primarily butyrate followed by propionate, inhibit the activation and expansion of the antigen-specific CTLs most probably by affecting the antigen-presenting DCs.

### Butyrate and propionate impairs up-regulation of co-stimulatory molecules on DCs

**Figure 2:**
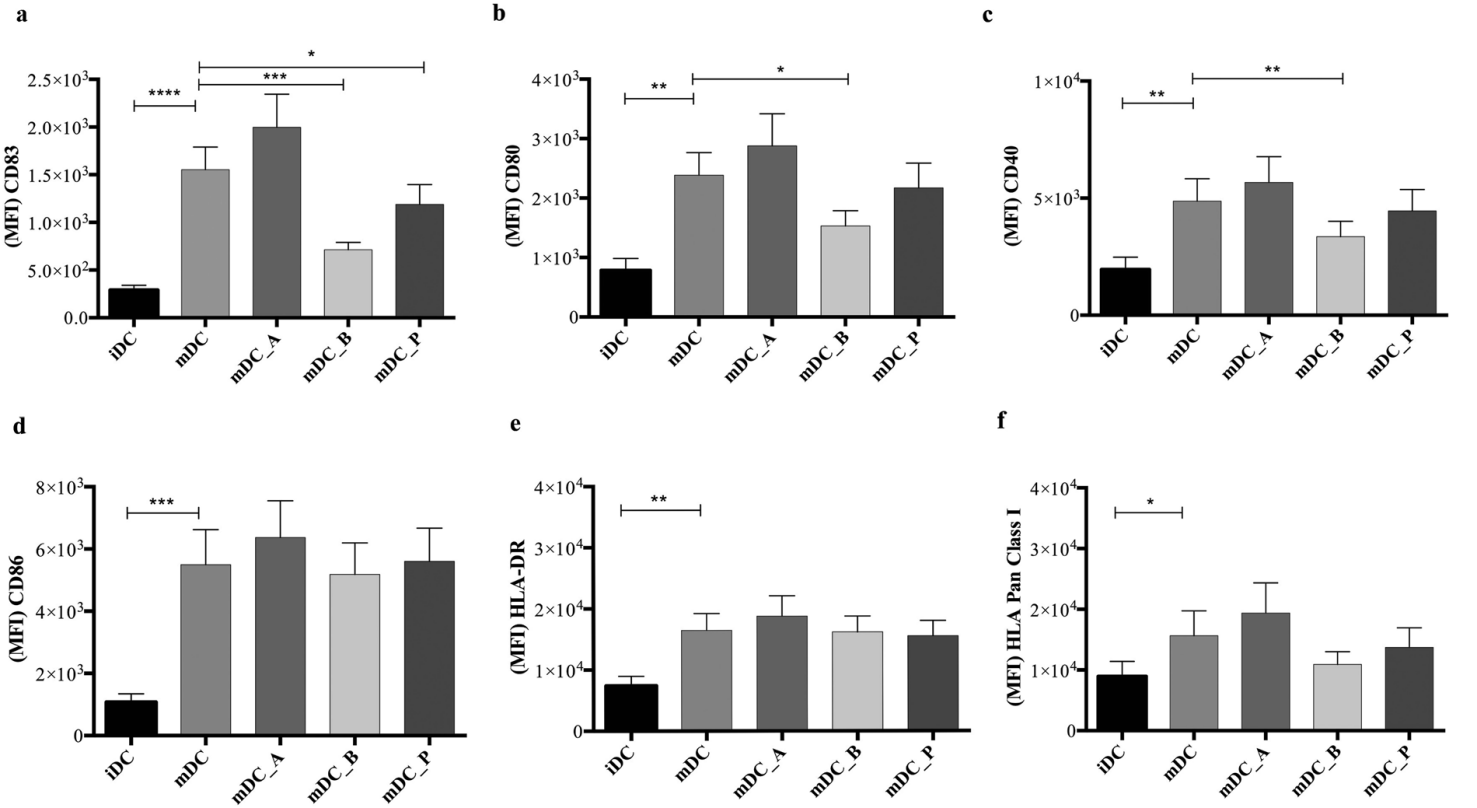
Butyrate and propionate impairs up-regulation of co-stimulatory molecules on DCs. Charts represent the mean fluorescence intensity (MFI) values of each donor’s sample. Shown are the averages ± standard deviations (SD) (a,b,f n = 14; e n = 12; c,b n = 8); Two-tailed paired Student’s t-tests, *P < 0.05, **P ≤ 0.01, ***P ≤ 0.001.

To examine how the individual SCFAs might affect DCs, we stimulated iDCs with LPS in the absence or presence of SCFAs and measured the surface expression of CD83, CD80, CD40, CD86, HLA-DR (MHC Class II), HLA-pan Class I (MHC Class I) by flow cytometry (Fig. 2a–f). We found a significant inhibition of the expression of the co-stimulatory molecules CD83, CD80, and CD40 after butyrate treatment (P ≤ 0.001, P ≤ 0.05, and P ≤ 0.01, respectively), and a significant reduction in CD83 and CD80 expression exerted by propionate (P ≤ 0.05, both) (Fig. 2a–c). SCFA treatment of mDCs had no effect on either surface expression levels of CD86, HLA-DR or HLA-ABC (Fig. 2d–f), or viability (Supplementary Fig. 3).

### SCFAs reduce the secretion of IL-6, IL-12, and IL-23 from DCs

**Figure 3:**
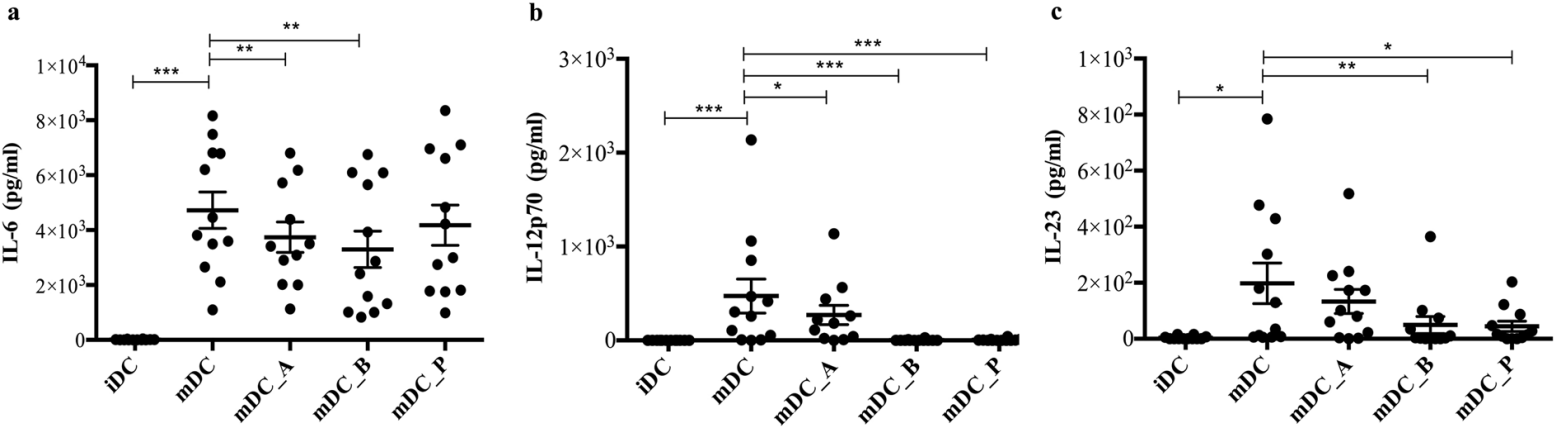
SCFAs reduce the secretion of IL-6, IL-12, and IL-23 from DCs. Shown are the averages ± standard deviations (SD) of (a) IL-6, (b) IL-12p40, (c) and IL-23 cytokines released into the media by monocyte-derived DCs and detected by ELISA from n = 12 donors. Two-tailed paired Student’s t-tests, *P < 0.05, **P ≤ 0.01, ***P ≤ 0.001.

As previously shown by our group, butyrate and propionate exert a strong immunomodulatory effect on LPS-stimulated DCs. Thus, we previously reported that butyrate and propionate-treated mDCs down-regulate LPS-induced IL-6, IL-12B, and chemokine genes and protein expression^19^. Here, we confirm what we have previously shown regarding IL-6 (Fig. 3a). In addition we have found that SCFAs affect the secretion of both IL-12p70 and IL-23 (n = 12) (Fig. 3b and c respectively). Indeed, butyrate and propionate strongly inhibit mDCs secretion of IL-12p70 (both P ≤ 0.001) (Fig. 3b) and IL-23 (P ≤ 0.01 and P ≤ 0.05, respectively) (Fig. 3c). We have previously shown that acetate had no effect on any of the investigated cytokines or chemokines^19^, but surprisingly we see a reduction of IL-12p70 secretion by mDCs after acetate treatments (P ≤ 0.05) (Fig. 3b).

### Addition of exogenous IL-12 rescues expansion of CTLs in co-cultures with SCFA-treated DCs

**Figure 4:**
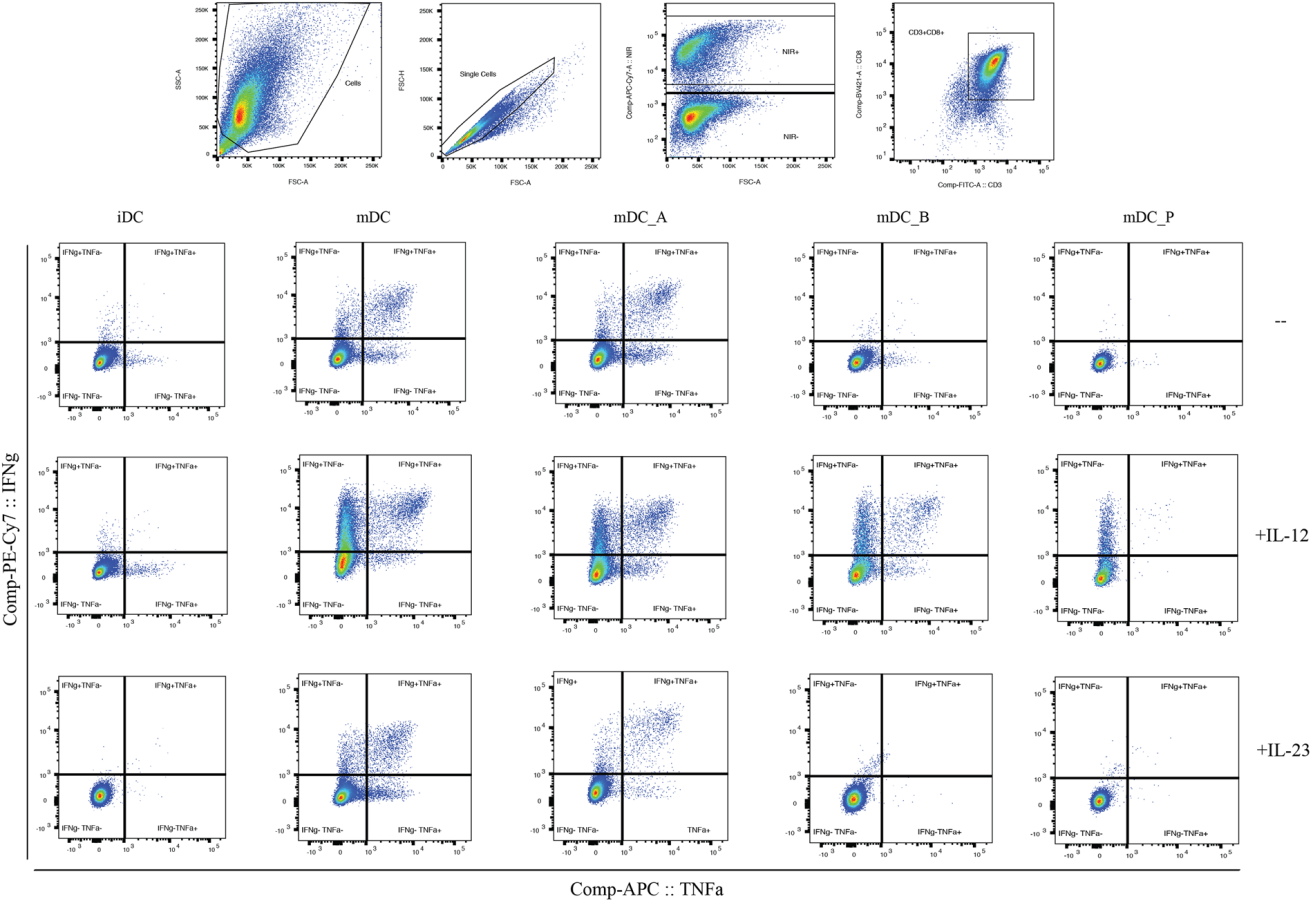
Gating strategy used for flow cytometry analysis. FACS plots are shown as representative example of the gating strategy used in each experiment, combined with the plots showing the comparison of IFN-γ and TNF-α cytokine production by CD8^+^ T cells after supplementing the media with eiter IL-12 or IL-23.

**Figure 5:**
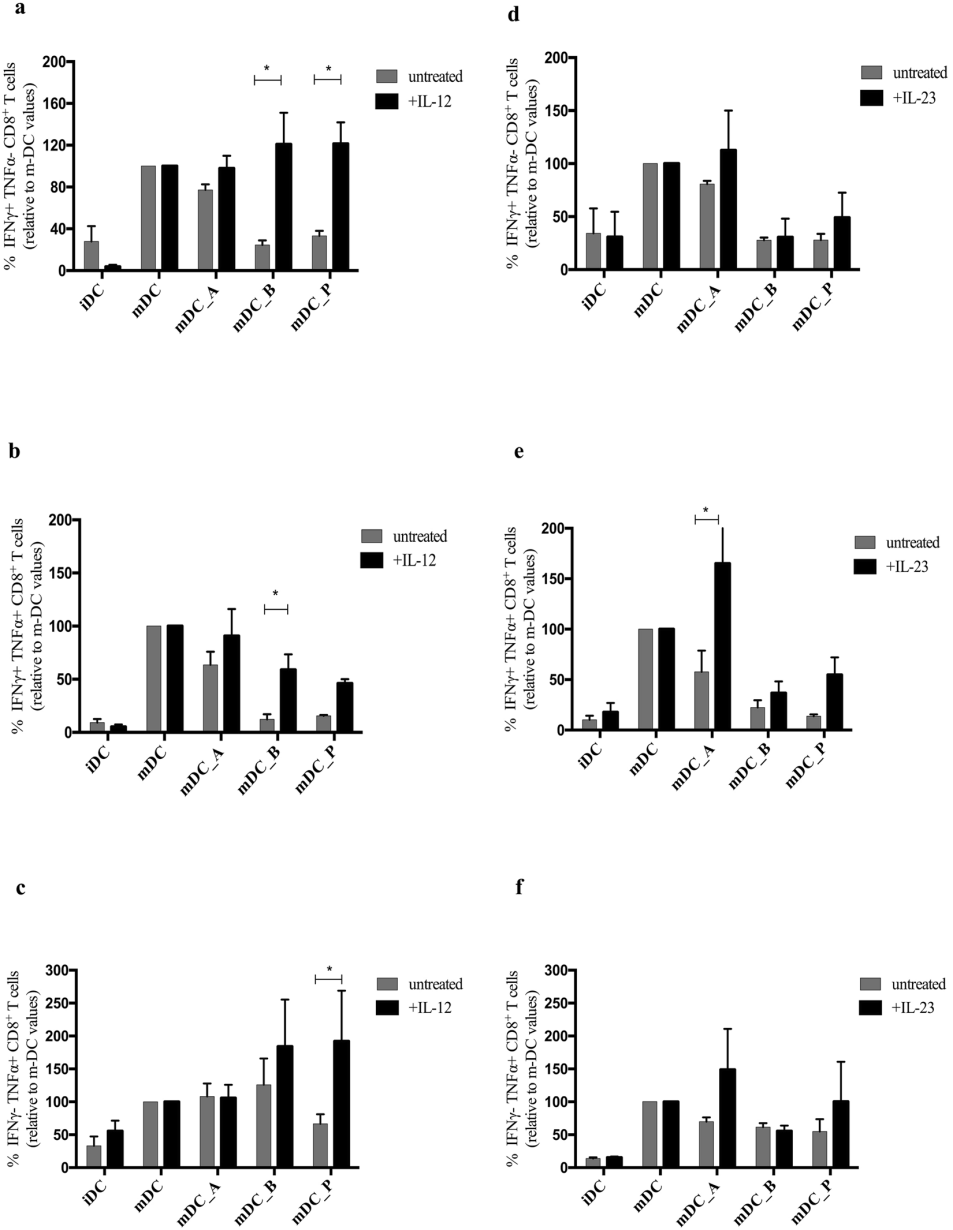
Addition of exogenous IL-12 rescues the activity of CTLs in co-cultures with SCFA-treated DCs. Charts represent the percentages of activated IFN-γ^+^ TNF-α^−^, IFN-γ^+^ TNF-α^+^, and IFN-γ^−^ TNF-α^+^-producing CD8^+^ T cells after 10 days of co-culture with MART-1 pulsed iDCs, mDCs, mDC_A, mDC_B, and mDC_P with and without (a–c) IL-12 (donors n = 5), (d–f) IL-23 (n = 3) supplementation into the media. Values have been normalized to those CD8^+^ T cells in co-culture with untreated mDC that has defined the activation threshold for each individual donor. Multiple t-tests with Holm-Sidak as correction method, *P < 0.05.

The inhibition of IL-6, IL-12p70 and IL-23 release from mature DCs and their subsequent lack in the surrounding niche led us to explore the presumable necessity to replace signal 3 for a CTLs activation. To this purpose we performed the same co-culture experiments and ICS analysis in parallel, as previously reported above, but now supplementing the culture media with each of the individual cytokines. As shown in Figs 4 and 5a, we observed that the exogenous IL-12 media supplementation was able to rescue the number of the IFN-γ^+^ TNF-α^−^ -producing CD8^+^ T cells population with butyrate- and propionate-treated mDC co-cultures (Fig. 5a, both P ≤ 0.05). The same has been observed in the raw data reported in Supplementary Fig. 5a, where it is also possible to appreciate the effect of IL-12 in significantly restoring the IFN-γ^+^ TNF-α^−^-producing CD8^+^ T cells in all three SCFA-treated mDC co-cultures (Supplementary Fig. 5a, all P ≤ 0.05).

Regarding the IFN-γ^+^ TNF-α^+^-producing CD8^+^ T cells population, IL-12 seemed to exert the same effect only on those ones in co-culture with butyrate-treated mDCs (n = 5) (Fig. 4 and 5b, P ≤ 0.05). This unfortunately can not be appreciated in the raw data reported in Supplementary Fig. 5b. About the IFN-γ^−^TNF-α^+^-producing CD8^+^ T cells population, IL-12 seemed to rescue them only if in co-cultures with propionate-treated mDC (Figs 4 and 5c, P ≤ 0.05). On the other hand, neither the addition of IL-23 (n = 3) (Figs 4 and 5d–f) nor of IL-6 (n = 2) (Supplementary Fig. 4) was capable to restore the CTLs functionality, indicating that the butyrate and propionate affect the CD8^+^ T cell mainly via the inhibition on DCs production of IL-12. Furthermore, IL-12 reintroduction into the culture can sufficiently drive back the CTLs functionality to a normal level, overcoming the lack of costimulatory molecules. It has previously been shown that IL-12, but not IL-23, significantly augments IFN-γ production by CD8^+^ T cells^34^ and that IL-12 provides the essential signal 3 for the naïve CD8^+^ T cells priming and activation by DCs^28^.

Taken together, our results provide a new insight to how SCFA affect DCs ability to prime CTLs and induce their cytokines production, and shead new light to the understanding of the exclusive and strong effect of IL-12.

## Discussion

The microbiome plays an important role in health and disease^1,2,35^ and a shift in balance between a healthy and dysbiotic microbiome leads to pathologic and metabolic conditions including obesity, colon cancer, IBD, and allergic asthma^17,36–39^. Short-chain fatty acids, such as acetate, butyrate and propionate, are end-products of microbial fermentation of macronutrients by the human gut microbiota that distribute systemically via lymphatics to the blood^5,6^. It is well known that SCFAs show immunomodulatory abilities, exerting a specific effect on distinct human and murine immune cells^3,13,19,40,41^. Antigen-presenting cells, such as DCs, couple innate recognition to adaptive immunity and, thus, the signals that convert resting antigen-presenting cells into immunogenic, or tolerogenic, are crucial. In fact, a successful priming of the adaptive immune responses is directly dependent on the innate signals and stimuli used to activate the innate component. SCFAs have shown to exert a important role in directly shaping the naive CD4^+^ T cells polarization, either directly or via their effect on DCs^40,42^. In this study we tested a hypothesis that SCFA, via their effect on DCs, can also impact the priming of CD8^+^ T cell, and thus their responses.

Impairing DCs maturation or any of the activating signals leads to a reduced functionality of the effector cells of the adaptive immune system including CD8^+^ T cells^43^. DCs deliver the required signals to activate naïve CD8^+^ T cells, and without these signals the adaptive immune responses would not be elicited. DCs ability to stimulate naïve T cells strictly depends on their state of maturation^20^. Thus, mature DCs will present several key activating ligands or secreted factors to naïve CD8^+^ T cells during priming such as peptide-MHC complexes (signal 1), costimulatory molecules (signal 2), and cytokines (signal 3)^30^. Generally, expression of costimulatory molecules on DC is essentially involved in controlling T cell differentiation and the resulting immune responses. We observed that both butyrate and propionate down-regulate the LPS-induced co-stimulatory molecules CD83, CD80, and CD40 presumably inhibiting their ability to correctly interact with CD8^+^ T cells during the priming and activation steps. MHC class I/II expression is pivotal for activation of T cells providing the first signal needed for the activation of a naïve T cells. We observed no effect of SCFA on MHC class I or II expression, indicating that SCFA do not compromise the antigens presentation via MHC molecules, thus a correct interaction between TCR and MHC can still be established. CD80 and CD86 expression on DCs probably constitute the most important costimulatory molecules in T cell activation. Signaling through binding partner CD28 on T cells confers optimal mRNA stabilization and production of IL-2, a factor that promotes expansion and survival of primary T cells^44^. Human iDC constitutively express intermediate amounts of CD86 and lack CD80, while mature DCs express higher amount of both^45^. While the down regulation of CD80 may compensate by the unaffected CD86 (considering their shared ligand), the lack of CD40 and CD83 might not. Indeed, it is known that the CD40 binds CD40L on T cells and its pathway regulates cellular and humoral immunity and plays an important role in T cell priming and differentiation^46^. Indeed, CD40 ligation on DCs increases expression of costimulatory, adhesion and MHC molecules and promotes the production of T cell stimulatory cytokines such as IL-12^47,48^. CD83 functions as an enhancer during the stimulation of T cells, and induces clear changes in cytokine expression during T cell priming^49^. Furthermore, the engagement of CD83 ligand on human T cells preferentially enriches, and significantly amplifies, the number of antigen-specific CD8^+^ T cells, thus by affecting its expression it results in compromised T cell proliferation^50–52^. We have observed the same tendency of butyrate-or propionate-treated mDC to decrease the numbers of IFN-γ^+^TNF-α^−^ and the IFN-γ^+^TNF-α^+^-producing CD8^+^ T cells when in co-culture in all experiments regardless of the peptides used (Fig. 1 and Supplementary Fig. 2); nevertheless we found a strong consistency of CD8^+^ T cell responses when MART-1 peptide was used compared to the others. CD8^+^ T cell responses to different antigen-derived peptides depend greatly on their affinity towards MHC class I, and to prior exposure to infections (EBV and CMV). This gives very individual responses to the different peptides, and result in a large donor variation. Taken together, this led us to consider MART-1 the most reliable peptide to use as a model for further analysis. Overall, we observed that both butyrate- and propionate-treated mDC were significantly impaired in priming/inducing the IFN-γ^+^ TNF-α^−^-producing-, IFN-γ^+^ TNF-α^+^-producing CD8^+^ T cells, and only propionate to affect the IFN-γ^−^ TNF-α^+^-producing CD8^+^ T cells, while the peptide-specificity and the proliferation of the CD8^+^ T cells were not impaired.

Taken together, our results indicate the the effects of butyrate and propionate on DCs ability to activate CD8^+^ T cells are not merely cause by the lack of CD40, CD80, and CD83 on the DCs surface (that leads to a reduction of survival cytokines release by the activated T cells e.g. IL-2, IL-5) but to a higher degree to the impairment of signal 3, that is usually represented by the cytokines produced by the antigen presenting cells.

We have confirmed, what we have previously shown, that butyrate significantly reduce IL-6 expression and release by human monocyte-derived DCs^19^. In addition, we here show that both butyrate and propionate down-regulates the LPS-induced cytokine release of IL-12p70 and IL-23; cytokines that polarize naïve CD4^+^ T cells towards Th1 and Th17, thus eliciting pro-inflammatory properties as recently shown by Haghikia *et al*.^42^. To investigate whether reduced expression of cytokines from SCFA-treated DCs were responsible for the observed CD8^+^ T cells activation, we performed co-culture experiments with supplemented cytokines. Indeed, when supplementing the co-culture media with IL-12 the IFN-γ^+^ TNF-α^−^-producing CD8^+^ T cells were restored, and even enhanced, in both butyrate- and propionate-mDC co-cultures; while the IFN-γ^+^ TNF-α^+^-producing CD8^+^ T cells population was just sufficiently restored in butyrate-mDC co-cultures, and the IFN-γ^−^ TNF-α^+^-producing CD8^+^ T cells population was just rescued in the propionate-mDC co-cultures. We did not observe the same effect with either IL-23 or IL-6 supplementation, leading to support the important role that IL-12 plays in the CD8^+^ T cells activation and in initiating and supporting the IFN-γ and TNF-α production. Indeed, as previously shown^28–30^, IL-12 provides a third signal exclusively to naive CD8^+^ T cells, and the response to IL-12 as well as to peptide antigens depends on co-stimulation. IL-12 is known to increase CD8^+^ T cell cytolysis, survival, proliferation, and at the same time influence the ability of the cells to migrate to inflammatory loci. On the other hand, IL-23 has the ability to stimulate the production of IL-17 by CD4^+^ T cells, important for immune reactions against extracellular pathogens^27^, but its role in the activation of naïve CD8^+^ T cells is still poorly understood. IL-12 and IL-23 share the p40 subunit, but most probably due to their specific and different receptors, they activate distinct sets of downstream transcriptor factors, thus that IL-12 leads to IFN-γ production while IL-23 does not induce neither IFN-γ nor TNF-α. In our study, we show that SCFAs affect co-stimulatory molecules surface expression on DCs, as well as cytokine production of these APCs and ability to prime CD8^+^ T cells. On the other hand SCFAs rather impair the CTLs IFN-γ^+^ and TNF-α^+^ production and peptide-specificity but have no effect on proliferation. Thus, we assumed that it could be due to the down-regulation of CD40, CD80 and particularly of CD83 expression (that indirectly leads to a reduced cytokines release by T cells as already shown by Prechtel *et al*.^49^), but also due to the impairment of signal 3 by treated DCs.

We here propose that SCFA, produced by our commensals, support a more tolerogenic activation of the innate immune system’s component rather than a pro-inflammatory stimulation. This could be seen as a strategic way to avoid a strong immune response towards their own producers, damping a potential cytotoxic activity in the gut that could impair the gut mucosa integrity, while not affecting the immune system’s ability to defeat a potential invading pathogen.

## Materials and Methods

### Peripheral blood mononuclear cells (PBMC) isolation

PBMCs were isolated from buffy coats obtained from anonymous healthy blood donors by Ficoll-Hypaque density gradient centrifugation. Before processing, all the buffy coats were screened for HLA-A0201 positivity using HLA-A2 (BD, BB7.2) antibody and only those positive were chosen for further analysis. Written informed consent was obtained from blood donors at the Department of Clinical Immunology, University Hospital Rigshospitalet, Copenhagen and used without the possibility to identify case specific information: The ethical committee, Region H, Capital Region of Denmark, approved the use of these buffy coats for research that was carried out in accordance with the approved guidelines.

### Culture of monocyte-derived DC and autologous CD8^+^ T cells

CD14^+^ monocytes and CD8^+^ T cells were isolated from PBMCs by positive selection using magnetic beads in accordance with the manufacturer’s instructions (Miltenyi Biotec, Germany). Monocytes were cultured at 37 °C in 5% CO_2_ in media supplemented with 10% fetal bovine serum (FBS, ThermoFisher) GM-CSF and IL-4 (both 50 ng/ml; PeproTech, Rocky Hill, NJ) for 7 days to generate iDC and mDC, the last obtained after LPS (100 ng/ml, *E*. *coli* 055:B5, Sigma-Aldrich) stimulation for the last 24 h in parallel with a single addition of 1 mM to acetate, propionate or butyrate (Sigma-Aldrich) leading to the following groups of treatments: sodium acetate (mDC_A), sodium butyrate (mDC_B), sodium propionate (mDC_P).

### DC-CD8^+^ T cells co-culture

After 7 days of culture, iDCs, mDCs, mDC_A, mDC_B, and mDC_P were harvested, the supernatants stored, and cells were washed and resuspended in X-VIVO 15 (Gibco, Lonza), and subsequently pulsed with 1 mM MART-1 peptide (ELAGIGILTV), EBV BLMF1 peptide (GLCTLVAML), PDL101 peptide (LLNAFTVTV), or CMV pp65 peptide (NLVPMVATV) (all by KJ Ross Petersen Aps, Copenhagen, Denmark) at 37◦C for 2 h. Hereafter DCs (0.5 × 10^5^) were washed twice and incubated in 48-wells culture plates together with autologous CD8^+^ T cells (0.5 × 10^6^). The general informations and throubleshooting advices were taken from Wolfl *et al*.^53^. For each condition (iDCs, mDCs, mDC_A, mDC_B, mDC_P) were plated 3–5 co-culture replica in order to be able to exclude possible technical errors. At day 3 and 6, half of the medium was refreshed with new containing rIL-2 (50 U/ml, Novartis) and rIL-7 (10 ng/ml, Peprotech, Rocky Hill, NJ). For experiments with supplement of exogenous rIL-12, rIL-6 and rIL-23 (10 ng/ml; Peprotech, Rocky Hill, NJ) were added together with the medium refreshment at day 3 and 6 of the co-culture.

### Flow cytometry analysis

Cells were stained at 4 °C for 30 min in FACS buffer (1%BSA, 0.01% sodium azide, PBS), washed, resuspended in the same buffer. Isotype-matched antibodies were used to define and exclude the background staining. 7-AAD (BD Pharmigen) (for the DCs phenotype), or LIVE/DEAD® Fixable Far Near-IR stain (Invitrogen) (for the intracellular stainings) was used to exclude dead cells. DCs analysis was conducted with anti-human: HLA-pan class I (clone W6/32), HLA-DR (clone L243), anti-human CD83 (clone HB15e), CD86 (clone 2331 FUN1), CD40 (clone 5C3), CD80 (clone L307.4), all purchased at BD Bioscience.

Functional analysis of CD8^+^ T cell from co-culture experiments were preformed via intracellular cytokine stainings at day nine. CD8^+^ T cells were harvested, washed and plated in a 96-wells round plates, and re-stimulated with MART-1 peptide for 4 h with the addition of GolgiPlug (Brefeldin A, BD) for the last 3 h. Anti-human CD3 (BD, clone SK7), CD8 (BD, clone RPA-T8) and LIVE/DEAD® Fixable Far Near-IR stain (Invitrogen) were used for gating live CD3^+^CD8^+^ T cells, followed by the fixation and the permeabilization with the Transcription factor Staining Buffer Set (eBioscience, 00–5523) for the detection analysis of intracellular cytokines production by anti-human IFN-γ (clone B27) and TNF-α (MAb11) (both BD Bioscience). For analysis of antigen-specificity, CD8^+^ T cells stained with PE-conjugated peptide-HLA-A2 MHC-tetramer (30 nM, immunAware, Denmark) for 30 min at 37 °C followed by surface markers staining of CD8 and CD3 for 30 min at 4 °C. Data acquisition and flow cytometric analysis were done on by LSRFortessa™ (BD Bioscience) at the CFFC (Core Facility for Flow Cytometry, Faculty of Health and Medical Sciences, University of Copenhagen). All data analysis was performed with FlowJo v7.0 (Treestar, Ashland, OR).

### ELISA assay

Supernatants from DC maturation cultures were collected and frozen at 80 °C during DC harvest. IL-6, IL-12p70, and IL-23 production was measured in the supernatants of DC by ELISA following the manufacturer’s instructions (R&D Systems, Inc.).

### Statistical analysis

Two-tailed paired Student’s t-tests were used with a significance level of 0.05. Before each test the Gaussian distribution of the samples data was checked though D’Agostino-Pearson normality test, if the test was not passed a non-parametric test was performed. All the analysis were made by GraphPad Prism 6.0 and the nomenclature for the P values is the following: *P ≤ 0.05, **P ≤ 0.01, ***P ≤ 0.001, ****P ≤ 0.0001.

Each donor has been considered as independent experiment and all the flow cytometry data were performed with 3 up 5 technical replicates for each condition.

## Electronic supplementary material

Supplementary Information 
