# Supplementary material for: Butyrate and propionate inhibit antigen-specific CD8+ T cell activation by suppressing IL-12 production by antigen-presenting cells
Source: Sci Rep. 2017 Nov 6;7:14516. doi: 10.1038/s41598-017-15099-w (PMC5673935; doi:10.1038/s41598-017-15099-w)
Supplement: Supplementary file 1 — Supplementary Information [file 41598_2017_15099_MOESM1_ESM.doc]

**Butyrate and propionate inhibit antigen-specific CD8^+^ T cell activation by suppressing IL-12 production by antigen-presenting cells**

Claudia Nastasi^1^, Simon Fredholm^1^, Andreas Willerslev-Olesen^1^, Morten Hansen^2^, Charlotte Menné Bonefeld^1^, Carsten Geisler^1^, Mads Hald Andersen^1,2^, Niels Ødum^1^ and Anders Woetmann^1^

^1^Department of Immunology and Microbiology, University of Copenhagen, Denmark; ^2^Center for Cancer Immune Therapy (CCIT), Department of Hematology, Copenhagen University Hospital, Herlev, Denmark;

Corresponding author:

Anders Woetmann, Department of Immunology and Microbiology, University of Copenhagen, Copenhagen, Denmark. E-mail: awoetmann@sund.ku.dk

**
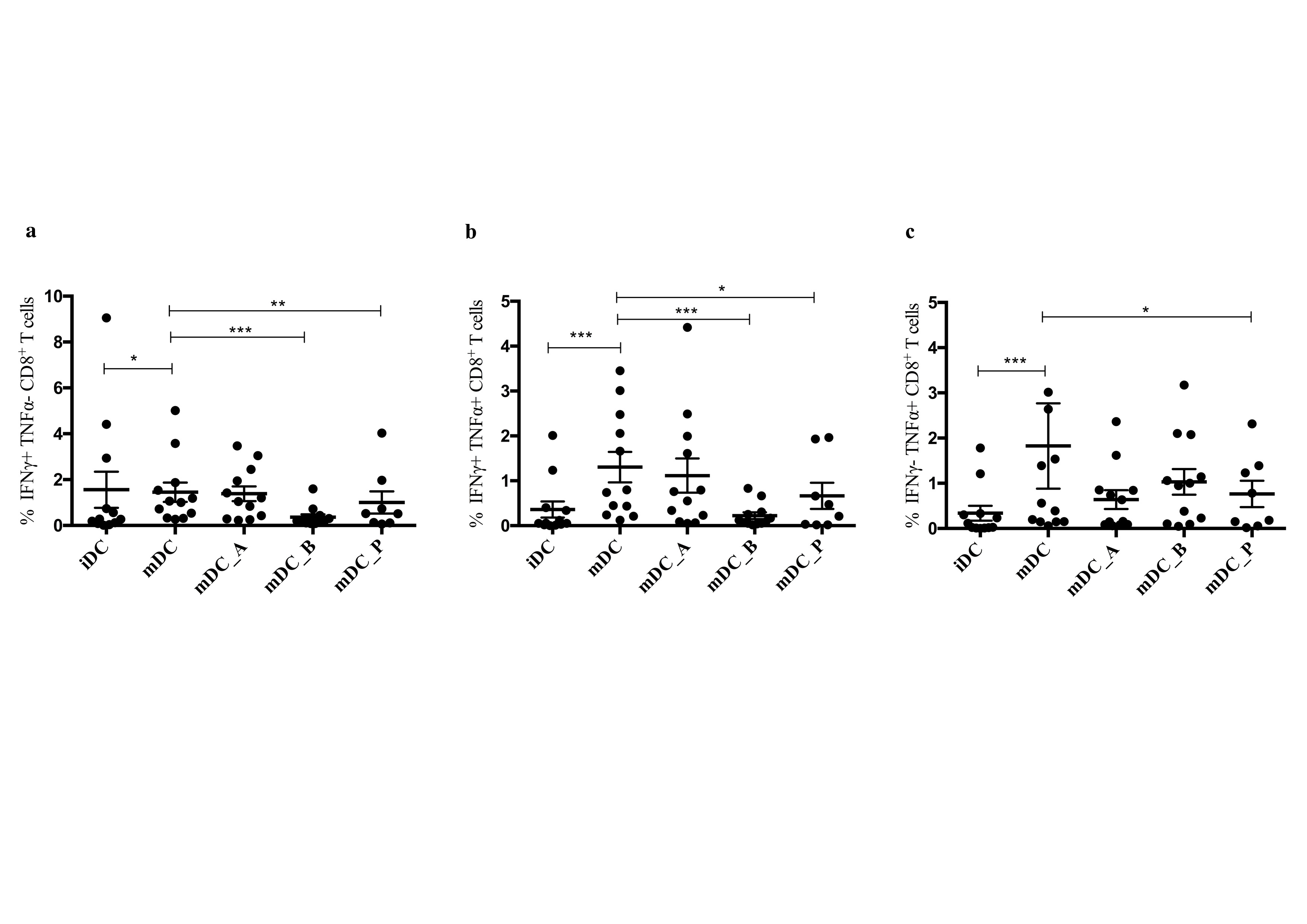
**

**Supplementary figure 1. Raw data of MART-1-specific CTLs in co-cultures with SCFA-treated DCs.** Chart represents the raw percentages of activated (**a**) IFN-γ^+^ TNF-α^-^-, **(b)** IFN-γ^+^ TNF-α^+^-, and (**c**) IFN-γ^-^ TNF-α^+^-producing CD8^+^ T cells after 10 days of co-culture with MART-1 pulsed iDCs, mDCs, mDC_A, mDC_B, and mDC_P, (donors n=12). Wilcoxon matched-pairs signed rank tests, *P<0.05 ***P ≤ 0.001.

**
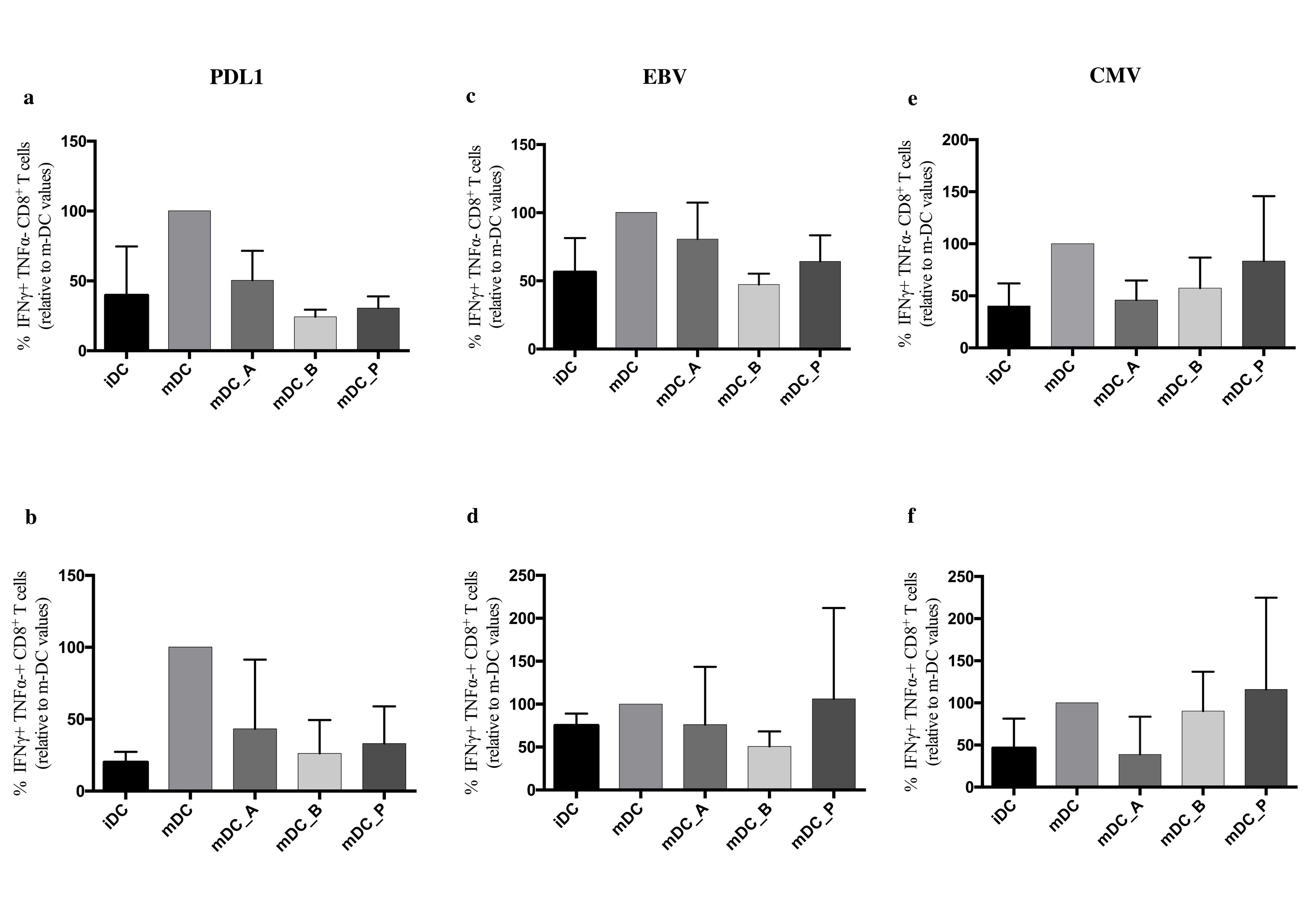
**

**Supplementary Figure 2. Butyrate- and propionate-treated mDC reduce the activation of CTLs towards several peptides.** Chart represents the percentages of activated IFN-γ^+^ TNF-α^-^- and IFN-γ^+^ TNF-α^+^- producing CD8^+^ T cells after 10 days of co-culture with PDL1 (**a-b**) (n=2), EBV (**c-d**) (n=4), or CMV (**e-f**) (n=4) pulsed iDCs, mDCs, mDC_A, mDC_B, and mDC_P. Values have been normalized to those CD8^+^ T cells in co-culture with untreated mDCs that has defined the activation threshold for each individual donor.

**
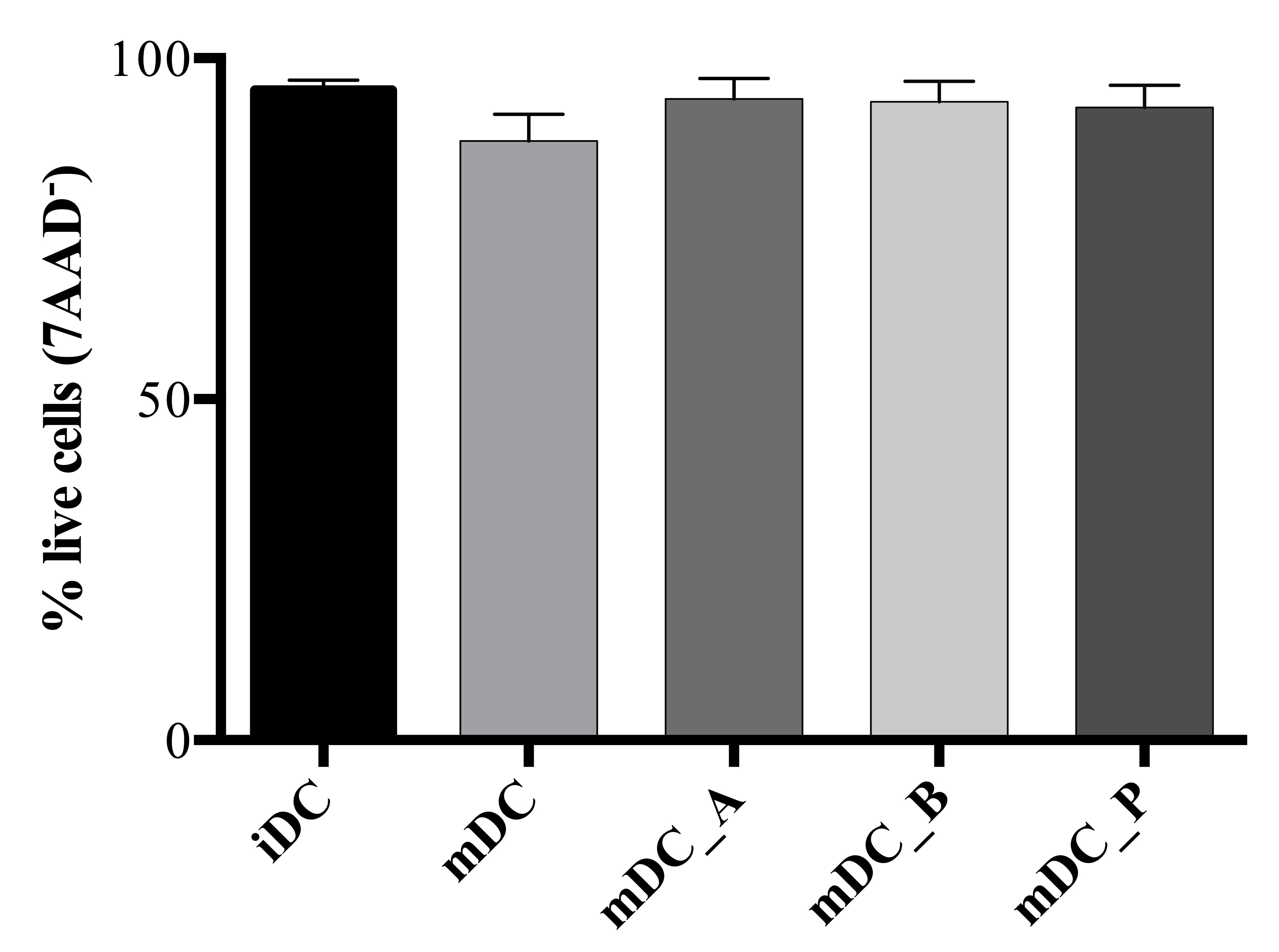
**

**Supplementary figure 3. SCFAs do not affect dendritic cells viability.** The chart shows the percentages of live SCFAs treated-DCs cells as negative to 7AAD staining assessed by flow cytometry. Shown are the averages ± standard deviations (SD).

**
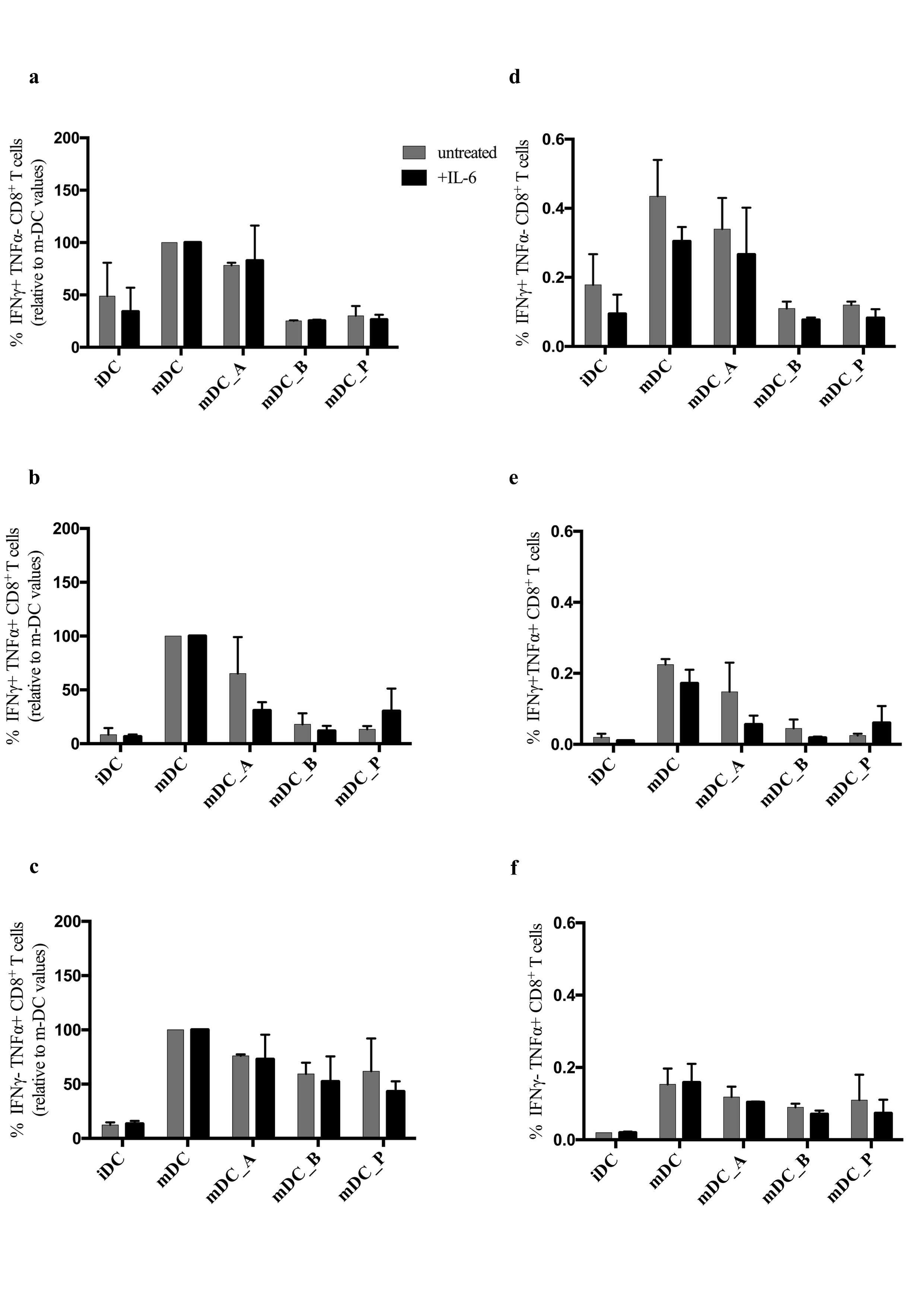
**

**Supplementary figure 4. Addition of exogenous IL-6 do not affect the activity of CTLs in co-cultures with SCFA-treated DCs.** Charts represent the percentages of activated IFN-γ^+^ TNF-α^-^- (**a**), IFN-γ^+^ TNF-α^+^- (**b**), and IFN-γ^-^ TNF-α^+^- (**c**) producing CD8^+^ T cells after 10 days of co-culture with MART-1 pulsed iDCs, mDCs, mDC_A, mDC_B, and mDC_P with and without IL-6 (donors n=2) supplementation into the media. In the left panel values have been normalized to those CD8^+^ T cells in co-culture with untreated mDC that has defined the activation threshold for each individual donor, in the right panel the correspondent raw values are shown.

**
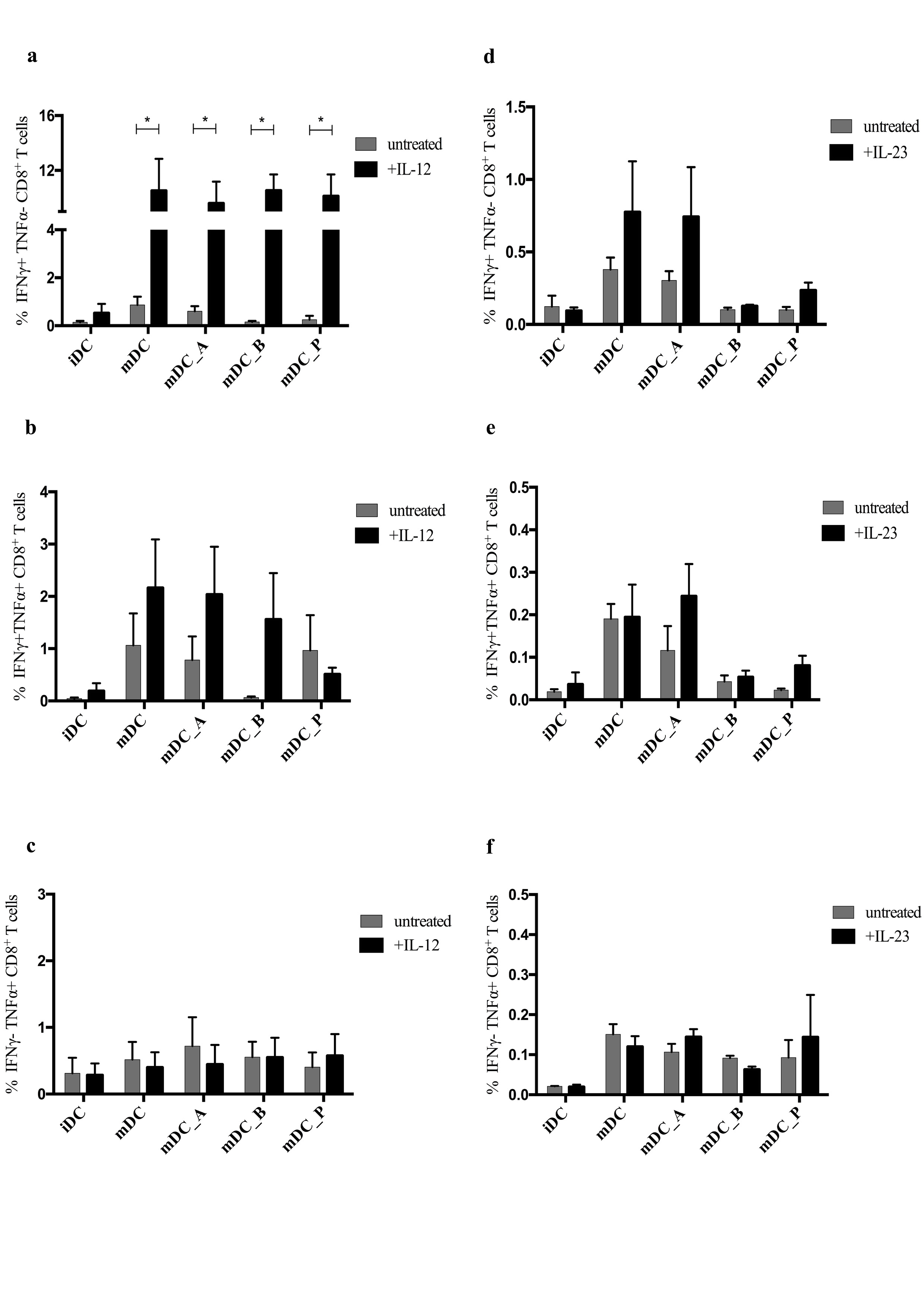
**

**Supplementary figure 5. Raw data of MART-1-specific CTLs in co-cultures with SCFA-treated DCs with IL-12 and IL-23 supplementation** Shown in parallel are the charts representing the raw percentages of the three CTLs populations supplemented with IL-12 (donors n=5) (**a-c**), or IL-23 (n=3) (**d-f**). Multiple t-tests with Holm-Sidak as correction method, *P<0.05.
